# Clinical and Pathological Predictors of Death for Adrenocortical Carcinoma

**DOI:** 10.1210/jendso/bvad170

**Published:** 2024-01-08

**Authors:** Eduardo Pato, Victor Srougi, Claudia Zerbini, Felipe L Ledesma, Fabio Tanno, Madson Q Almeida, William Nahas, Ana Claudia Latronico, Berenice B Mendonca, Jose L Chambô, Maria Candida B V Fragoso

**Affiliations:** Division of Urology, Hospital das Clínicas, University of São Paulo School of Medicine, São Paulo, SP 05403-010, Brazil; Division of Urology, Hospital das Clínicas, University of São Paulo School of Medicine, São Paulo, SP 05403-010, Brazil; Division of Pathology, Hospital das Clínicas, University of São Paulo School of Medicine, São Paulo, SP 05403-010, Brazil; Division of Pathology, Hospital das Clínicas, University of São Paulo School of Medicine, São Paulo, SP 05403-010, Brazil; Division of Urology, Hospital das Clínicas, University of São Paulo School of Medicine, São Paulo, SP 05403-010, Brazil; Division of Endocrinology, Hospital das Clínicas, University of São Paulo School of Medicine, São Paulo, SP 05403-010, Brazil; Division of Urology, Hospital das Clínicas, University of São Paulo School of Medicine, São Paulo, SP 05403-010, Brazil; Division of Endocrinology, Hospital das Clínicas, University of São Paulo School of Medicine, São Paulo, SP 05403-010, Brazil; Division of Endocrinology, Hospital das Clínicas, University of São Paulo School of Medicine, São Paulo, SP 05403-010, Brazil; Division of Urology, Hospital das Clínicas, University of São Paulo School of Medicine, São Paulo, SP 05403-010, Brazil; Division of Endocrinology, Hospital das Clínicas, University of São Paulo School of Medicine, São Paulo, SP 05403-010, Brazil

**Keywords:** adrenocortical carcinoma, hormonal hypersecretion, risk factors, mortality

## Abstract

Adrenocortical carcinoma (ACC) is a rare and lethal disease with a poor prognosis. This study aims to share our 41-year experience as a referral center, focusing on identifying risk factors associated with ACC mortality. Our retrospective analysis included a cohort of 150 adult patients with ACC in all stage categories, treated between 1981 and 2022. Tumor hormonal hypersecretion was observed in 78.6% of the patients, and the median age of diagnosis was 40 years. The majority presented as European Network for the Study of Adrenal Tumors (ENSAT) III or IV (22.9% and 31.2%, respectively), and the overall mortality rate was 54.6%. Independent predictors of death were elevated secretion of cortisol (HR = 2.0), androstenedione (HR = 2.2), estradiol (HR = 2.8), 17-OH progesterone (HR = 2.0), and 11-deoxycortisol (HR = 5.1), higher Weiss (HR = 4.3), modified Weiss (HR = 4.4), and Helsinki scores (HR = 12.0), advanced ENSAT stage (HR = 27.1), larger tumor size (HR = 2.7), higher Ki-67 percentage (HR = 2.3), and incomplete surgical resection (HR = 2.5). Mitosis greater than 5/50 high-power field (HR = 5.6), atypical mitosis (HR = 2.3), confluent necrosis (HR = 15.4), venous invasion (HR = 2.8), and capsular invasion (HR = 2.4) were also identified as independent predictors of death. Knowing the risk factors for ACC's mortality may help determine the best treatment option.

Adrenocortical carcinoma (ACC) is a rare disease, with annual incidence ranging from 0.7 and 2 cases per million [1, 2]. This tumor is more frequent in women and has a peak incidence in the fourth and fifth decade of life [3]. The prognosis often is unfavorable, with an overall survival time of 1.7 years [4, 5]. The main treatment option for localized tumors is adrenalectomy, which should be performed by an experienced surgeon using open, laparoscopic, or robotic-assisted techniques [5, 6]. However, roughly 50% of patients are diagnosed with locally advanced or metastatic disease. In such cases, the current treatment options have limited efficacy. The role of radiotherapy is still under debate, and the objective response rate for first-line chemotherapy is 23% [7]. New treatments, such as immunotherapy, are under investigation but currently offer precarious outcomes.

Prognostic risk factors were assessed to guide therapeutic choices and help to improve survival. The European Network for the Study of Adrenal Tumors (ENSAT) staging system is accepted worldwide and uses the same features as the American Joint Committee on Cancer [8]. Histologic parameters also have an essential role. The Weiss (original and modified) and the Helsinki scores measure the tumor grade and determine the prognosis [9]. Genetic testing may be the next trend in risk assessment, but there is no consensus on gene profile and applicability to date. The most known mutation associated with ACC is at the *TP53* gene, which may be related to worst prognosis [3, 10-13].

Considering the rarity and lethality of ACC and the precarious results of the available treatments, identifying risk factors can facilitate personalized treatment strategies that may improve outcomes. This study aims to report our 42-year experience as a referral tertiary center in Brazil and identify the risk factors for mortality associated with ACC.

## Materials and Methods

We retrospectively analyzed 150 patients diagnosed with ACC treated at the University of São Paulo Medical School between 1981 and 2022 (after institutional review board approval). Patients in all staging groups were included. The exclusion criteria were patients younger than 18 years and harboring other primary tumors. The diagnosis of ACC was confirmed pathologically by 2 experienced pathologists (in the surgical specimen or adrenal lesion biopsy) or by a typical clinical presentation for ACC in unresectable tumors (excess adrenal hormone, adrenal tumor ≥4 cm, and distant metastasis in imaging studies).

Demographic (age and sex) and clinical parameters (asymptomatic, Cushing syndrome, virilization syndrome, and ENSAT stage) were retrieved. We have evaluated the hormonal profile of our patients by measuring cortisol (through late-night salivary cortisol, serum cortisol following the dexamethasone 1-mg suppression test, and the 24-hour urinary free cortisol level), aldosterone, dehydroepiandrosterone, androstenedione, testosterone, 17-OH progesterone, 11-deoxycortisol, 11-deoxycorticosterone, and estradiol. Hormone excess was considered if any of these hormones were increased. The hypercortisolemia diagnosis was considered if the cortisol was elevated in one test and confirmed on a second examination.

Surgical specimens retrieved before 2017 were reviewed by the pathologists for Ki-67 analysis and Weiss reclassification. Among 126 operated patients, 122 had the specimen available for reassessment. We assessed the tumor and treatment characteristics as follows: percentage of Ki-67, TP53 expression, lymphovascular invasion, Helsinki score, original and modified Weiss score, as well as its isolated parameters (nuclear grade 3 or 4, >5 mitoses per 50 high-power field, presence of atypical mitoses, clear cell component at 25% of tumor or more, diffuse architecture at one-third of tumor or more, confluent necrosis, venous invasion, sinusoidal invasion, and capsular infiltration), tumor size, laterality, type of resection (when realized) and use of adjuvant mitotane, chemotherapy, or radiotherapy.

For the analysis, we have categorized some continuous variables as follows: tumor size up to 12 cm and higher than 12 cm; Ki-67 up to 20% and higher than 20%; Weiss score up to 5 and higher than 5; modified Weiss score up to 4 and higher than 4; Helsinki score up to 12, 13 to 20, and higher than 20.

The primary end point was the occurrence of death by any cause. Survival time was defined from the date of the first image examination to report an adrenal tumor to the date of death or last consultation, since our cohort includes patients treated clinically, and therefore we could use the pathological analysis date as a landmark. All retrieved variables were assessed to evaluate their association as risk factors for mortality. The quantitative variables were described using mean and SD, while qualitative variables were described using percentage and frequency. The association of the measured parameters and death was analyzed with the parametric *t* test for variables with homogeneous distribution, nonparametric Mann-Whitney for variables with heterogeneous distribution, and chi-square test for categorical variables. Cox regression was used to evaluate the relationship between variables and outcomes, and Kaplan-Meier curves were used to analyze survival outcomes. Univariate analysis was performed to identify risk factors for mortality. Logistic regression was performed for the significant associations found in the univariate analysis; hazard ratios (HRs) were calculated. Kaplan-Meier curves were built for survival estimates. Statistical significance was set at *P* less than .05. The statistical software SPSS 22.0 was used for the analysis.

## Results

Demographic, clinical, and hormonal data are shown in Tables 1 and 2. There was no difference in age, sex, or laterality between the alive and the deceased. The deaths by ENSAT stage 1, 2, 3, and 4 were 1 (7.7%), 17 (32.7%), 21 (65.6%), and 39 (88.6%), respectively. A total of 118 patients (78.6%) had hormonal hypersecretion. The *TP53* mutation in our cohort was present in 12 patients (8%) but was not associated with death (*P* = .22). The median follow-up was 33.5 months (interquartile range, 18.2-91.2 months).

**Table 1.** Demographic features

Total No.	150
Median age at diagnosis (IQR)	40 (30-54)
Sex	45 M (30%)
	104 F (70%)
Laterality	78 L (53.1%)
	69 R (46.9%)
Tumor size median in cm (IQR)	11.6 (8-20)
Hormonal hypersecretion	118 (78.6%)
Surgical resection	126 (84%)
Positive margins	20 (21.9%)
ENSAT stage	13 I (9.1%)
	53 II (36.8%)
	33 III (22.9%)
	45 IV (31.2%)
Median follow-up in mo (IQR)	33.5 (18.2-91.2)
Deaths	82 (54.6%)

Abbreviations: ENSAT, European Network for the Study of Adrenal Tumors; F, female; IQR, interquartile range; M, male.

**Table 2.** General endocrinological features

Parameter	Yes/Altered	No/Normal
Cushing syndrome	86 (65.6%)	45 (34.4%)
Women with high androgen	55 (61.8%)	34 (38.2%)
Men with high estrogen	7 (17.9%)	32 (82.1%)
High cortisol level	101 (71.1%)	41 (28.9%)
High mineralocorticoid	10 (8.1%)	114 (91.9%)
High aldosterone	10 (10.2%)	88 (89.8%)
High DHEAS	48 (44%)	59 (54.1%)
High androstenedione	45 (45.9%)	53 (54.1%)
High testosterone	43 (40.9%)	62 (59%)
High 17-OH progesterone	30 (33.7%)	59 (66.3%)
High estradiol	16 (16.5%)	81 (83.5%)

Abbreviation: DHEAS, dehydroepiandrosterone sulfate.

On univariate analysis, the following parameters were significantly associated with death: clinical Cushing syndrome (*P* = .034), female patients with elevated androgen (*P* = .03), high cortisol (*P* = .023), high androstenedione (*P* = .001), high 17 OH-progesterone (*P* = .002), high 11 deoxycortisol (*P* < .001), high estradiol (*P* = .017), high DHEAS (dehydroepiandrosterone sulfate) (*P* = .048), larger tumor size (*P* < .001), presence of distant metastasis (*P* < .001), ENSAT stage (*P* < .001), type of resection (*P* = .033), clear cell component less than or equal to 25% (*P* = .008), mitoses greater than 5/50 high-power field (HPF) (*P* < .001), confluent necrosis (*P* < .001), venous invasion (*P* = .001), lymphovascular invasion (*P* = .007), tumor recurrence (*P* < .001), Ki-67 percentage (*P* = .001), both Weiss (*P* < .001) and modified Weiss score (*P* < .001), and Helsinki score (*P* < .001).

**Figure 1.**
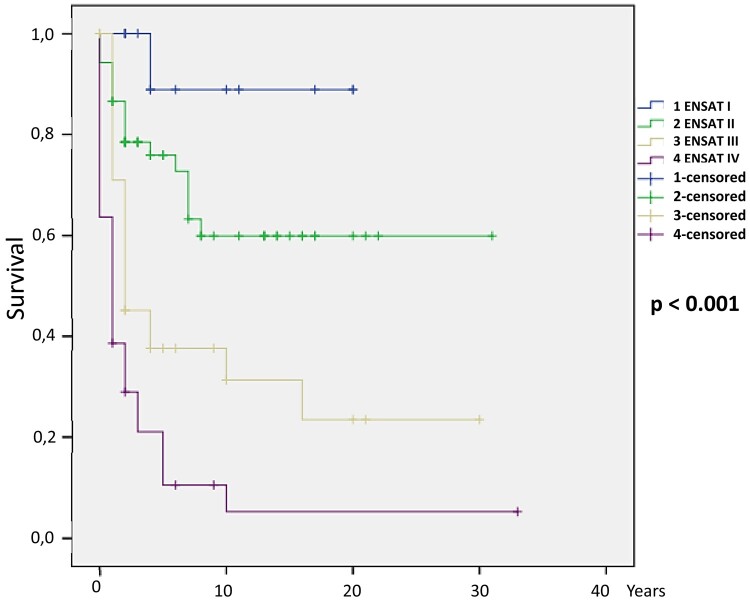
ENSAT stage Kaplan-Meier survival curve: Higher ENSAT stage is associated with mortality in our cohort (*P* < .001).

**Figure 2.**
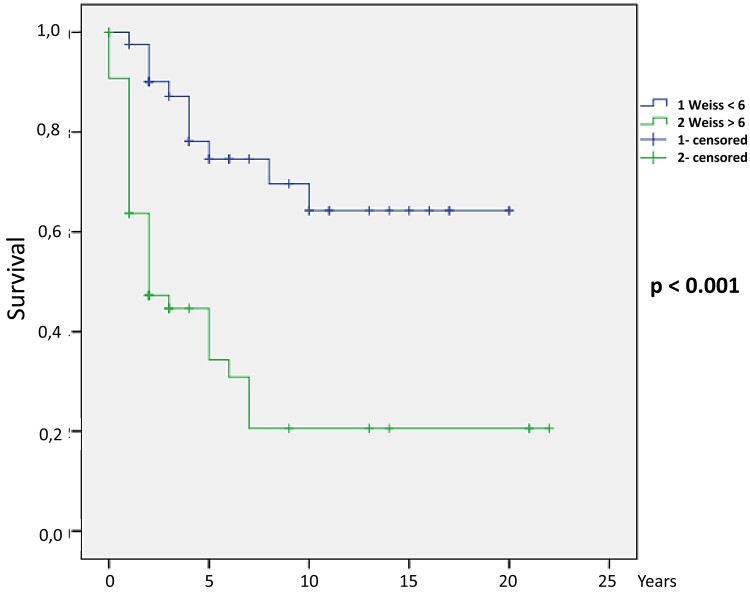
Weiss score Kaplan-Meier survival curve: Higher Weiss score is associated with mortality in our cohort (*P* < .001).

**Figure 3.**
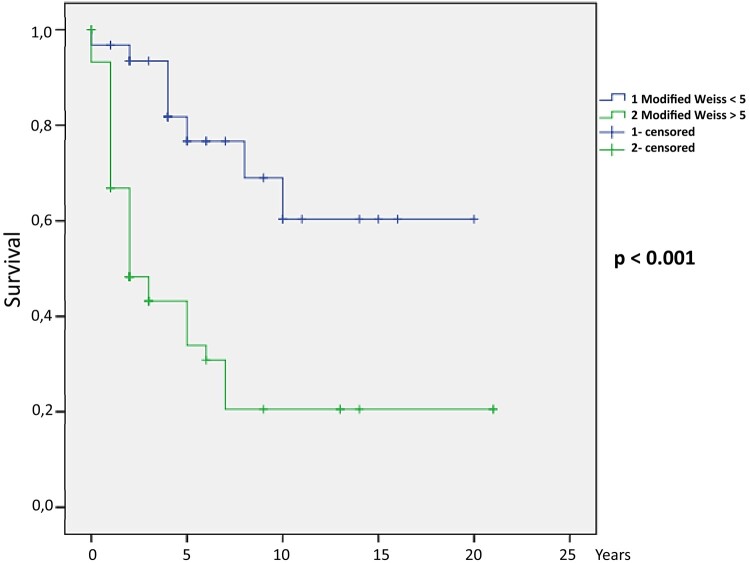
Modified Weiss score Kaplan-Meier survival curve: Higher modified Weiss score is associated with mortality in our cohort (*P* < .001).

**Figure 4.**
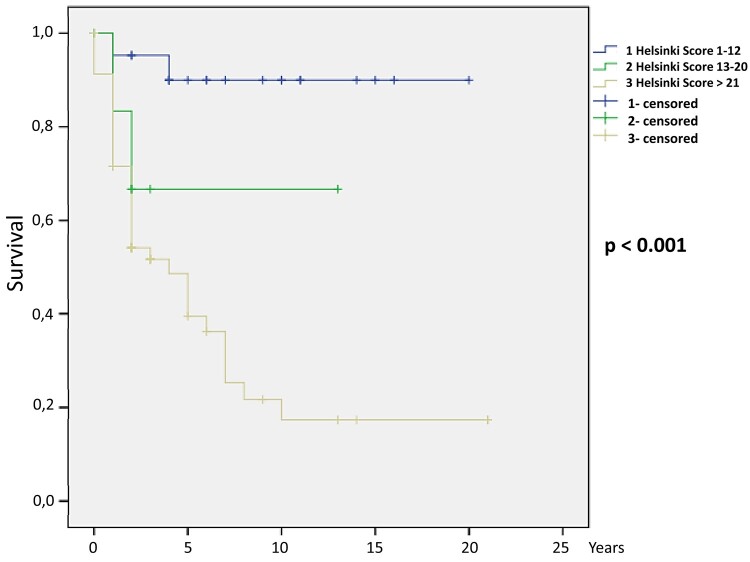
Helsinki score Kaplan-Meier survival curve: Higher Helsinki score is associated with mortality in our cohort (*P* < .001).

After logistic regression, the following parameters were independent predictors of death: clinical Cushing syndrome (*P* = .036), female patients with elevated androgen (*P* = .043), high cortisol (*P* = .014), high androstenedione (*P* = .006), high 17 OH-progesterone (*P* = .021), high 11 deoxycortisol (*P* = .001), high estradiol (*P* = .003), larger tumor size (*P* < .001), presence of distant metastasis (*P* < .001), ENSAT stage III (*P* = .015) and IV (*P* = .001), mitoses greater than 5/50 HPF (*P* < .001), atypical mitoses (*P* = .005), confluent necrosis (*P* = .007), venous invasion (*P* < .001), capsular infiltration (*P* = .006), lymphovascular invasion (*P* = .002), Ki-67 percentage (*P* = .001), tumor recurrence (*P* < .001), both Weiss (*P* < .001) and modified Weiss score (*P* < .001), and Helsinki score (*P* = .002). The HRs for each parameter are shown in Table 3. The Kaplan-Meier curves for ENSAT stage (Fig. 1), Weiss score (Fig. 2), modified Weiss score (Fig. 3), and Helsinki score (Fig. 4) are shown next.

**Table 3.** Independent predictors of death by adrenocortical carcinoma

Parameter	HR	95% CI
Cushing syndrome	1.8	1.0-3.0
Women with high androgen	0.5	0.3-0.9
High cortisol	2.0	1.1-3.4
High androstenedione	2.2	1.2-3.8
Estradiol		
Increased	2.8	1.1-5.6
Decreased	5.6	1.7-18.5
High 17-OH progesterone	2.0	1.1-3.5
High 11-deoxycortisol	5.1	1.9-13.1
Tumor size	2.7	1.7-4.3
Distant metastasis	4.9	3.0-7.8
ENSAT stage at diagnosis		
1		
2	4.6	0.6-34.3
3	11.9	1.6-88.8
4	27.1	3.7-197.7
Mitoses > 5/50 HPF	5.6	2.5-12.7
Atypical mitoses	2.3	1.3-4.2
Confluent necrosis	15.4	2.2-111.6
Venous invasion	2.8	1.6-5.2
Capsular infiltration	2.4	1.3-4.4
Weiss score ≥ 5	4.3	2.2-8.6
Modified Weiss score ≥ 5	4.4	2.0-9.6
Lymphovascular invasion	2.7	1.5-5.0
Ki-67, %	2.3	1.4-3.8
Helsinki score		
<12		
13-20	5.5	0.8-39.3
>20	12.0	2.9-50.2
Surgical resection		
R0		
R1	2.5	1.3-4.8

Abbreviations: ENSAT, European Network for the Study of Adrenal Tumors; HPF, high-power field; HR, hazard ratio.

The 10-year survival estimates were calculated for ENSAT stage, Helsinki score, Weiss, and modified Weiss score, as follows: ENSAT stage 1, 2, 3, and 4 were 89.9%, 59.9%, 41.4%, and 10.6%, respectively (*P* < .001); Helsinki scores 1 to 12, 13 to 20, and 21 or more were 89.9%, 66.7%, and 21.8%, respectively (*P* < .001); Weiss less than 5 and 5 or greater were 69.6% and 20.6%, respectively (*P* < .001); and modified Weiss score less than 5 and 5 or greater were 69% and 20.6%, respectively (*P* < .001).

## Discussion

In a cohort comprising all stage categories, we found several risk factors associated with a poorer prognosis of ACC. The tumor grading and staging classifications were strongly associated with death, among the strongest predictors.

Despite the higher prevalence of ACC in women, the mortality rate between sexes was equivalent. Other demographic parameters also did not influence the prognosis. Libé et al [14] reported that patients older than 50 years with ACC have a 1.6-fold greater risk of death than younger patients. One may suppose that ACC manifests with more aggressiveness in older adults. Alternatively, older patients could be more fragile and have worse tolerance to the aggressive treatments used for ACC. Surprisingly, age was not a predictor of death in the present cohort.

Most of the studied population had hormonal hypersecretion, while historically, it is expected that 20% to 40% of adults harboring ACC have nonsecreting tumors [15, 16]. We believe this discrepancy is due to the underdiagnosis of hormonal hypersecretion outside a dedicated service. In an academic environment, patients will likely be screened with more examinations, collected under ideal conditions, and using more precise assays. This fact could have increased our detection rate of hypersecretion, even when patients had no clinical manifestation. Of note, aberrant hormone production is a known risk factor for death by ACC [14]. We found several hormone alterations to be predictors of death, with estradiol and 11-deoxycortisol being the most significant (HR > 5). We also observed that women with androgen secretion had a lower risk of death, representing a novel finding. One may hypothesize that this subset was diagnosed and treated earlier because of the virilization syndrome manifestation.

*TP53* mutation is a known risk factor for ACC, and its frequency is as high as 80% in the pediatric population harboring this malignancy [17]. Worldwide, the prevalence of *TP53* mutation in adult ACC patients is approximately 4%, while in our cohort, it was 8% [15, 18]. This difference might be due to a high frequency of *TP53* mutation in a specific southern Brazil population, affecting 0.3% of newborns [19]. As a referral center for ACC management in Brazil, patients from this region might be sent to be treated at our institution, explaining this finding. However, we did not find *TP53* to be associated with death. The median age of our cohort was 40 years, relatively younger than the ACC population of other studies performed on different continents [20]. Unrecognized differences in the genetic signature of ACC might explain this discrepancy, such as the higher frequency of *TP53*.

At diagnosis, 54% of our cohort had advanced disease, and most underwent surgery. Surgical resection in a metastatic scenario remains controversial, as insufficient robust evidence supports it [3]. Nevertheless, in our institution, we routinely perform cytoreductive surgery when technically feasible, and the patient's status performance is preserved. A multi-institutional paired analysis, the best evidence to date, demonstrated an improvement in overall survival when adopting this treatment strategy compared to patients treated solely with systemic therapy [21]. In our experience, most patients in the ENSAT 1 and 2 category were cured, and those in the ENSAT 3 and 4 category died, strengthening the concept that the only way to cure a patient with ACC relies on timely surgery. It also highlights that the current therapies for advanced ACC are inefficient. Conversely, our efforts should focus on early diagnosis and developing systemic drugs with new targets.

The modified Weiss system encompasses the 5 most significant pathological parameters associated with the risk of malignancy out of 9 of the original Weiss system. Both were originally described to differentiate benign from malignant adrenal tumors with similar accuracy [6, 22]. Later, they were reported to be associated with the worst prognosis, in accordance with our findings [14]. Among the original parameters, we found 5 to be predictors of death, confluent necrosis being the strongest (odds ratio = 15.4). Mitotic rate and Ki-67 percentage were also identified as prognostic markers. Both analyze cell proliferation and are part of different diagnostic scoring systems (Weiss system and Helsinki score). Considering our findings, pathologists should be encouraged to report all items of the Weiss system, not only to distinguish adenomas from carcinomas but also for prognostic purposes.

This study has inherited limitations of its retrospective nature. We should also consider the long study period during which the treatment modalities evolved. Patients with similar diseases in different timelines might have been treated with distinct therapies. The same two dedicated pathologists (C.Z. and F.L.L.) revised and classified all specimens to eliminate disparities in the pathological analysis created by time. Most ACC studies use the period between pathology assessment and last contact for survival analysis. Since our study population comprehends patients treated clinically and not all had surgical specimens, we used the date of the first image examination with an adrenal tumor as a landmark to define survival time. This could impair the comparison of our study to other cohorts. It should be emphasized that given the rarity of ACC, we assembled a substantial, large cohort with a comprehensive long-term follow-up. To our knowledge, the association of each Weiss parameter with death was described for the first time, which may influence the treatment choice.

## Conclusion

Our study demonstrates that hormonal hypersecretion, higher stages of disease, larger tumor size, and incomplete surgical resection are predictors of death by ACC. Pathological parameters of the Weiss score might also be used separately to predict prognosis. These findings might be used to tailor better treatment strategies.

## Data Availability

Some or all data sets generated during and/or analyzed during the current study are not publicly available but are available from the corresponding author on reasonable request.

## Abbreviations

ACC: adrenocortical carcinoma
ENSAT: European Network for the Study of Adrenal Tumors
HPF: high-power field
